# Preparation and Application of Sodium–Lanthanum Molybdate for the Photocatalytic Degradation of Coomassie Brilliant Blue G-250 Dye

**DOI:** 10.1021/acsomega.4c08777

**Published:** 2025-04-17

**Authors:** Caique D. A. Lima, Joyce A. Borges, Italo A. L. Santos, Angel A. Hidalgo, Josy A. Osajima, Adriel da Silva Almeida, Thiago M. B. F. Oliveira, Jefferson F. D. F. Araujo, Suellen D. T. de Barros, Marcelo E. H. Maia da Costa, Diego A. B. Barbosa, João V. B. Moura, Gardênia S. Pinheiro, Cleânio L. Lima

**Affiliations:** † Department of Physics, Campus Universitário Ministro Petrônio Portella, Universidade Federal do Piauí, Bairro Ininga, Teresina PI CEP: 64.049- 550, Brazil; ‡ Department of Physics, Pontifical Catholic University of Rio de Janeiro, Rua Marques de São Vicente, Rio de Janeiro 22451-900, Brazil; § Laboratório de Materiais Avançados − Limav, Campus Universitário Ministro Petrônio Portella, Universidade Federal do Piauí, Bairro Ininga, Teresina PI CEP: 64.049- 550, Brazil; ∥ Department of Chemistry, Campus Universitário Ministro Petrônio Portella, Universidade Federal do Piauí, Bairro Ininga, Teresina PI CEP: 64.049- 550, Brazil; ⊥ Science and Technology Center, Federal University of Cariri, Av. Tenente Raimundo Rocha, 1639, Cidade Universitária, Juazeiro do Norte 63048-080, CE, Brazil; # Department of Physics, Federal University of Maranhão, São Luís 65080-805, MA, Brazil

## Abstract

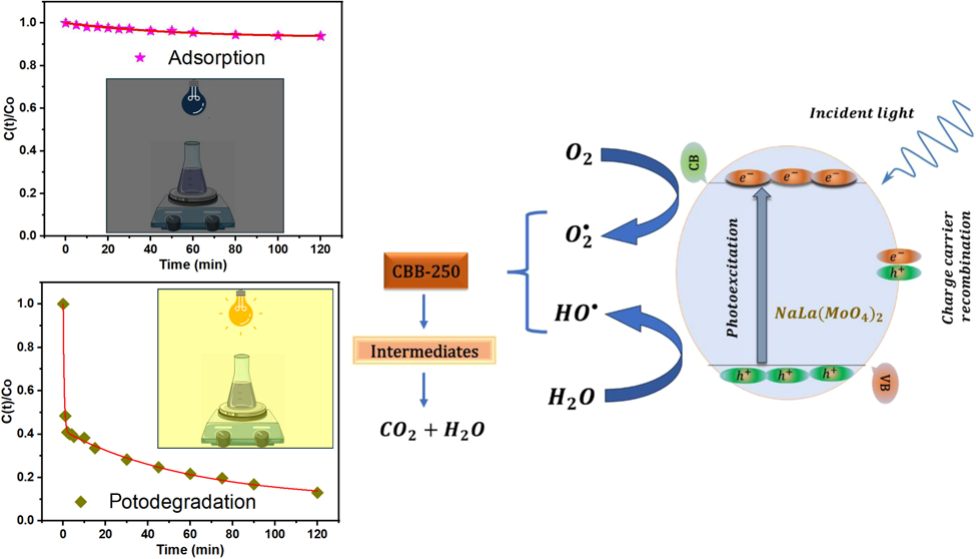

This study reports on the synthesis, characterization, and application of sodium–lanthanum molybdate [NaLa(MoO_4_)_2_] as a photocatalyst in the degradation process of Coomassie Brilliant Blue G-250 (CBB-250), an environmentally toxic and recalcitrant dye derived from triphenylmethane. The highlighted ceramic material was produced by coprecipitation, and its properties were studied using a range of physicochemical techniques including compositional, crystallographic, and morphological analyses. Among the most relevant information, X-ray diffraction (XRD), Raman spectroscopy, and Fourier transform infrared (FTIR) spectroscopy confirmed the formation of NaLa (MoO_4_)_2_ crystals with a tetragonal arrangement. Additionally, the scanning electron microscope (SEM) images provide evidence of the production of overlapping plate-like microstructures. The nitrogen adsorption isotherms demonstrated that the molybdate obtained exhibits mesoporous organization, with a surface area and pore diameter equivalent to 45.7 m^2^ g^–1^ and 6.54 nm, respectively. Photocatalytic tests, performed under ultraviolet light irradiation for 120 min, indicated that NaLa(MoO_4_)_2_ has high oxidative reactivity, achieving a degradation of >95% of the CBB-250 dye present in the system. The reaction velocity constants, estimated in accordance with the Langmuir–Hinshelwood equation, substantiate that NaLa(MoO_4_)_2_ enhances the reaction kinetics (13.7 × 10^–3^ min^–1^) throughout the photochemical treatment. In light of the growing scientific interest in effective strategies for the removal of emerging pollutants from contamination matrices, NaLa(MoO_4_)_2_ represents a promising avenue for the development of innovative technologies in this field.

## 1 Introduction

In recent years, there has been a significant amount of discourse surrounding environmental pollution, particularly the contamination of aquatic environments by substances, ions, and microorganisms with poorly understood side effects.^1−5^ The exacerbation of this issue can be attributed to a number of factors, including disordered population density, as well as unsustainable industrialization and agricultural processes. As a regrettable consequence, a considerable proportion of organic waste produced worldwide is released into the environment without any preliminary treatment, thereby compromising the living conditions of the exposed organisms.^6^

Synthetic dyes are among the compounds that cause high levels of pollution. These dyes are recalcitrant and present high levels of toxicity to ecosystems that are exposed to them. These compounds are utilized extensively across various industries, including textiles, pharmaceutical, cosmetic, plastic, automobile, paper, and food production.^7^ Dyes belonging to the triphenylmethane class, such as Coomassie Brilliant Blue G-250 (CBB-250), give rise to particular environmental concerns due to the lack of effective strategies for their adequate removal and/or treatment.^7−9^ (Photo)degradation processes are currently the most promising alternative procedure, and several ceramic materials are being tested for this purpose, including TiO_2_, CdS, ZnO, CeO_2_, and WO_3_, among others.^10−13^

While less extensively investigated, molybdates have also demonstrated potential as photocatalysts, offering a high surface area and remarkable chemical and thermal stability.^14−16^ Another significant benefit of these materials is the potential to coordinate multiple metallic centers within a single crystal lattice. This is exemplified by sodium–lanthanum molybdate [NaLa(MoO_4_)_2_], which allows for the alteration of their properties through doping or functionalization techniques, making them suitable for specific applications.^17,18^ The combination of rare-earth and alkali metals in a single ceramic composition represents an ongoing area of interest, with the objective of enhancing charge separation and augmenting photocatalytic activities.^18^ While lanthanides can act as dopants, introducing new energy levels for enhanced visible light absorption, alkali metals enhance ion mobility and improve electrical conductivity.^17,19^

A variety of synthesis routes are employed to obtain these compounds, including hydrothermal^18^ and solvothermal reactions,^20^ in addition to sol–gel,^21^ microemulsion,^22^ solid-state,^23^ and coprecipitation procedures.^24^ Coprecipitation is a particularly intriguing method due to its ability to produce homogeneous materials at relatively low temperatures with high yields.^19,24^ The objective of this study was therefore to assess the potential of NaLa(MoO_4_)2 as a photocatalyst for CBB-250 degradation, with a view of identifying new remediation solutions for matrices that are supposedly contaminated. To ascertain the efficacy of the synthesis and the photocatalytic activity of the compound throughout the degradation process, a series of physicochemical techniques were employed, utilizing a set of theoretical and experimental models that are well established in the literature. To ascertain the efficacy of the synthesis and the photocatalytic activity of the compound throughout the degradation process, a series of physicochemical techniques were employed, utilizing a set of theoretical and experimental models that have been well established in the literature.

## 2 Experimental Section

### 2.1 Molybdate Synthesis

The chemical coprecipitation method was employed to prepare NaLa(MoO_4_)_2_, using sodium molybdate dihydrate (Na_2_MoO_4_·2H_2_O) and lanthanum nitrate hexahydrate (La(NO_3_)_3_·6H_2_O) as the precursor materials. In the synthesis, 4.0 g of Na_2_MoO_4_.2H_2_O (99%, Sigma-Aldrich) were dissolved in 180 mL of deionized water and mixed under magnetic stirring for 20 min. The same procedure was followed to dissolve 4.0 g of La(NO_3_)_3_.6H_2_O (99%, Sigma-Aldrich) in 360 mL of deionized water. Subsequently, the solutions were meticulously combined and subjected to magnetic stirring for 4.0 h at a temperature of 80 °C. The precipitate resulting from this procedure was washed with deionized water and separated by centrifugation at 3600 rpm and dried at 298 K. The this synthesis route yielded 2.98 g of NaLa(MoO_4_)_2_,as evidenced by the following global reaction
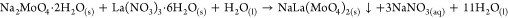
1

### 2.2 Characterization Studies

X-ray diffraction (XRD) patterns were obtained with a Shimadzu LABX-XRD 6000 powder diffractometer, utilizing Cu Kα radiation (λ = 1.5406 Å) over a 2θ range from 5 to 80° at a scan rate of 2°/min. The structural parameters of the samples were determined by the Rietveld method, utilizing the ReX software and the crystal data available in the ICSD (Inorganic Crystal Structure Database) code 1011043. Scanning electron microscopy (SEM) images were obtained using an FEI Quanta 250 FEG. The textural properties of the synthesized samples were determined from the nitrogen adsorption isotherms, which were recorded at 77 K on a Quantachrome NOVA 4200 apparatus. The specific areas were calculated by measuring the mass of the adsorbed nitrogen monolayer as a function of the relative pressure within the following equilibrium range: the pressure ratio was found to be between 0.0 and 1.0. The pore diameter was determined by the Brunauer–Emmett–Teller (BET) algorithm method, which was employed to analyze the adsorption and desorption isotherms. Raman spectra were obtained with a Bruker Senterra Raman spectrometer, which was coupled with an Olympus BX50 microscope and a charge-coupled device (CCD) image sensor detector. Additionally, the Raman spectrometer was also outfitted with an argon laser at 532 nm, with an output power of 5 mW. Fourier transform infrared (FTIR) spectroscopy experiments were conducted using a Bruker Vertex 70 spectrophotometer in the spectral range of 400–4000 cm^–1^, employing the transmission mode, and a pellet of a mixture of potassium bromide (KBr) and sample. The ultraviolet (UV)–visible absorption spectra were recorded with a UV-3600 spectrophotometer from Shimadzu. X-ray photoelectron spectroscopy (XPS) was performed using a hemispherical analyzer from SPECS model Phoibos 150, and the X-ray excitation energy was an Al ka monochromatic source (1486.6 eV) and flood gun with 1.7 μA and 5.0 V. The complex sample powder was fixed to the sample holder with a copper double tape. The pass energy for the survey was set at 50 eV, and other spectra were set at 20 eV, respectively. The C 1s peak at 284.8 eV was used as a reference. The spectra were deconvoluted in CasaXPS software using a pseudo-Voigt function with Gaussian–Lorentzian, 40% Lorentzian, and a Shirley-type background.

### 2.3 Photodegradation Experiments

The efficiency of NaLa(MoO_4_)_2_ and its precursor, Na_2_MoO_4_·2H_2_O, was evaluated as a photocatalyst for CBB-250 degradation under controlled conditions. The photocatalytic system was assembled in a closed chamber with a 125 W mercury lamp, with the reaction flask placed in a thermal bath at 298 K. The glass contained 0.2, 0.5, 0.75, and 1.0 g L^–1^ of the catalyst dispersed in an aqueous solution with 3.5 × 10^–5^ mol L^–1^ CBB-250. The solution was irradiated for 120 min while being stirred at 400 rpm. The degradation of the dye was observed at 0, 1, 2, 3, 4, 5, 7, 10, 15, 30, 45, 60, 75, 90, and 120 min of reaction time. Subsequently, the samples were subjected to centrifugation at 3500 rpm for 3 min, with the objective of reducing the interference caused by nonsupernatant particles. A kinetic study of the photocatalytic process was conducted based on the UV–vis data, with a focus on the time and rate of dye degradation. The kinetic model employed was originally described by Lagmuir and Hinshelwood and enables the calculation of the rate constant (*k*). The rate of degradation was calculated using the initial and equilibrium concentrations recorded throughout the reaction process. Although these experiments showed high stability, they were all performed in triplicate, and the results reported are averages of the values found.

In two supplementary experiments, the standard solution (0.5 g/L) underwent an adsorption and a degradation process. Photoluminescence (PL) and pH measurements were conducted at regular intervals of 0, 3, 5, 10, 20, 40, and 60 min. A third experiment involved UV–visible analysis of the standard dye solution (150 mL) at pH values of 4, 3, and 2, adjusted by using a 4 M hydrochloric acid solution. The initial solution, devoid of dye, exhibited a pH of 6. In a separate aqueous NaLa(MoO_4_)_2_ solution (0.5 g/L), PL and pH measurements were also performed, with recorded pH value of 6. Photoluminescence analyses were carried out using a PC1 photon-counting spectrometer with laser excitation at 317 nm, and emission was recorded in the range of 340 to 600 nm, corresponding to the dye absorption region.

## 3 Results

### 3.1 Structural and Vibrational Parameters

**Figure 1:**
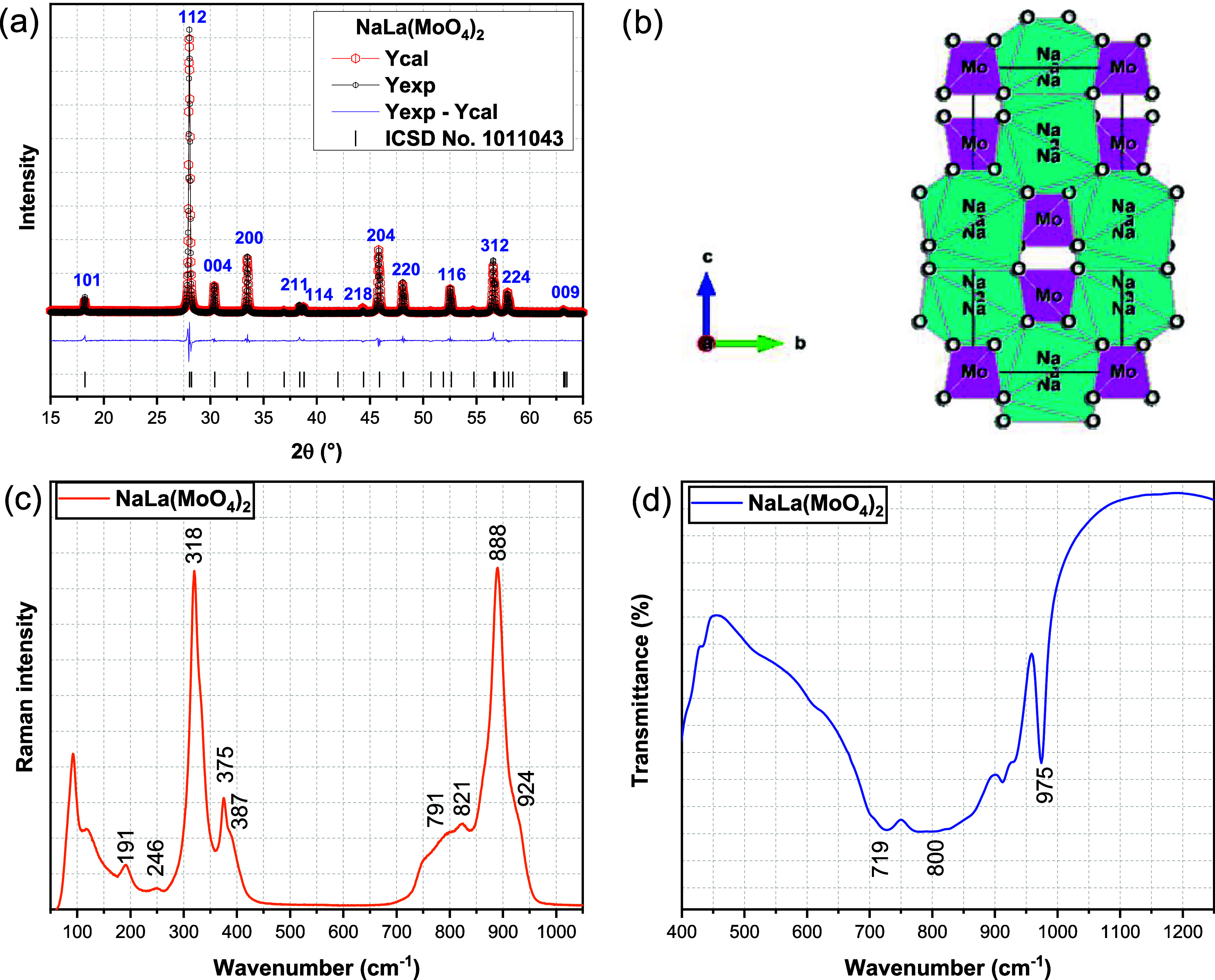
(a) XRD pattern, (b) unit cell perspective, and (c) Raman and (d) FTIR spectra of the NaCe(MoO_4_)_2_ microcrystals obtained by the coprecipitation method.

XRD analysis was employed to examine the structural arrangement and crystallinity of the as-obtained NaLa(MoO_4_)_2_ precipitate obtained in the previous step. An evaluation of the X-ray patterns illustrated in Figure 1a indicates that the recorded peaks can be attributed to the tetragonal phase, which exhibits a maximum at 3430 Å and *a* = *b* = 5.3537 and *c* = 11.7430 Å (JCPDS No. 24-1103) (Figure 1b).^25−27^ No other phase was identified. The thickness and intensity of the planes observed in the diffractogram indicate that NaLa(MoO_4_)_2_ exhibits good crystallinity. Figure 1b illustrates a unit cell of NaLa(MoO_4_)_2_ for reference, with Mo^6+^ ions surrounded by four oxygen atoms, resulting in the formation of [MoO_4_] clusters with a tetrahedral configuration. In contrast, the sodium and lanthanum ions coordinate with eight oxygen atoms, forming clusters with a dodecahedral structure and composition [NaLa_8_].

The structural features of NaLa(MoO_4_)_2_ remain a topic of speculation. In order to gain further insight, Raman spectra were recorded, and a representative result can be seen in Figure 1c. The spectra exhibited well-defined peaks that were indicative of tetragonal structures with a high degree of short-range ordering. The peaks at 869, 897, 912, and 977 cm^–1^ were identified as symmetric stretching modes, while those at 729, 773, and 823 cm^–1^ were identified as asymmetric stretching modes. Furthermore, the peaks at 328, 354, and 380 cm^–1^ were identified as corresponding to the asymmetric and symmetric bending modes of MoO_4_^2–^ tetrahedrons.^28^ The peaks at 236, 260, and 298 cm^–1^ were identified as translation and lattice vibrations.^28^ These findings are consistent with those observed through XRD, which also revealed long-range ordering.

In regard to the FTIR spectrum of the samples, as illustrated in Figure 1d, the band observed at 719 cm^–1^ was identified as the asymmetric stretching vibration, (MoO_4_^2–^), while those at 771 and 975 cm^–1^ were attributed to the symmetric stretching vibration of (MoO_4_^2–^).^28^ The results presented here demonstrate the formation of NaLa(MoO_4_)_2_ without the presence of a secondary phase.

**Figure 2:**
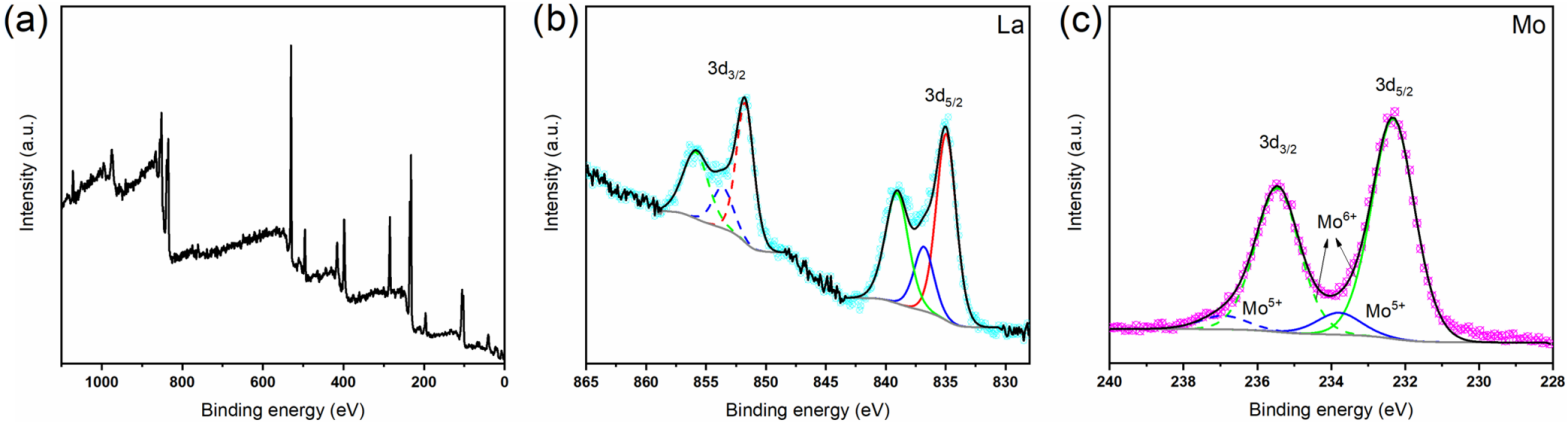
XPS spectra of (a) NaLa(MoO_4_)_2_ and peak decomposition of (b) La 3d and (c) Mo 3d.

X-ray photoelectron spectroscopy (XPS) of La_2_(MoO_4_)_3_ is shown in Figure 2. Figure 2a confirms the presence of Mo, La, O, and Na, while the Mo 3d and La 3d orbitals are shown in Figure 2b,c, respectively. Analysis of the d-orbitals for these two elements reveals two components, 3/2 and 5/2, separated by energy differences of 3.18 eV for Mo 3d and 16.98 eV for La 3d. For the La spectrum (Figure 2c), the peaks at 834.9, 839.1, 851.8, and 855.9 eV correspond to the La 3d_5/2_ and La 3d_3/2_ orbitals, characteristic of La^3+^.^29^ For molybdenum, analysis of the XPS shows that the Mo 3d region can be effectively modeled by two 3d peaks, corresponding to molybdenum in two different oxidation states. The primary peak, at 232.4 and 235.5 eV, is characteristic of Mo^6+^, while the secondary peak, centered at 233.8 and 236.9 eV, corresponds to Mo^5+^.^29−31^

### 3.2 Topographic Profile and Photoactivity

**Figure 3:**
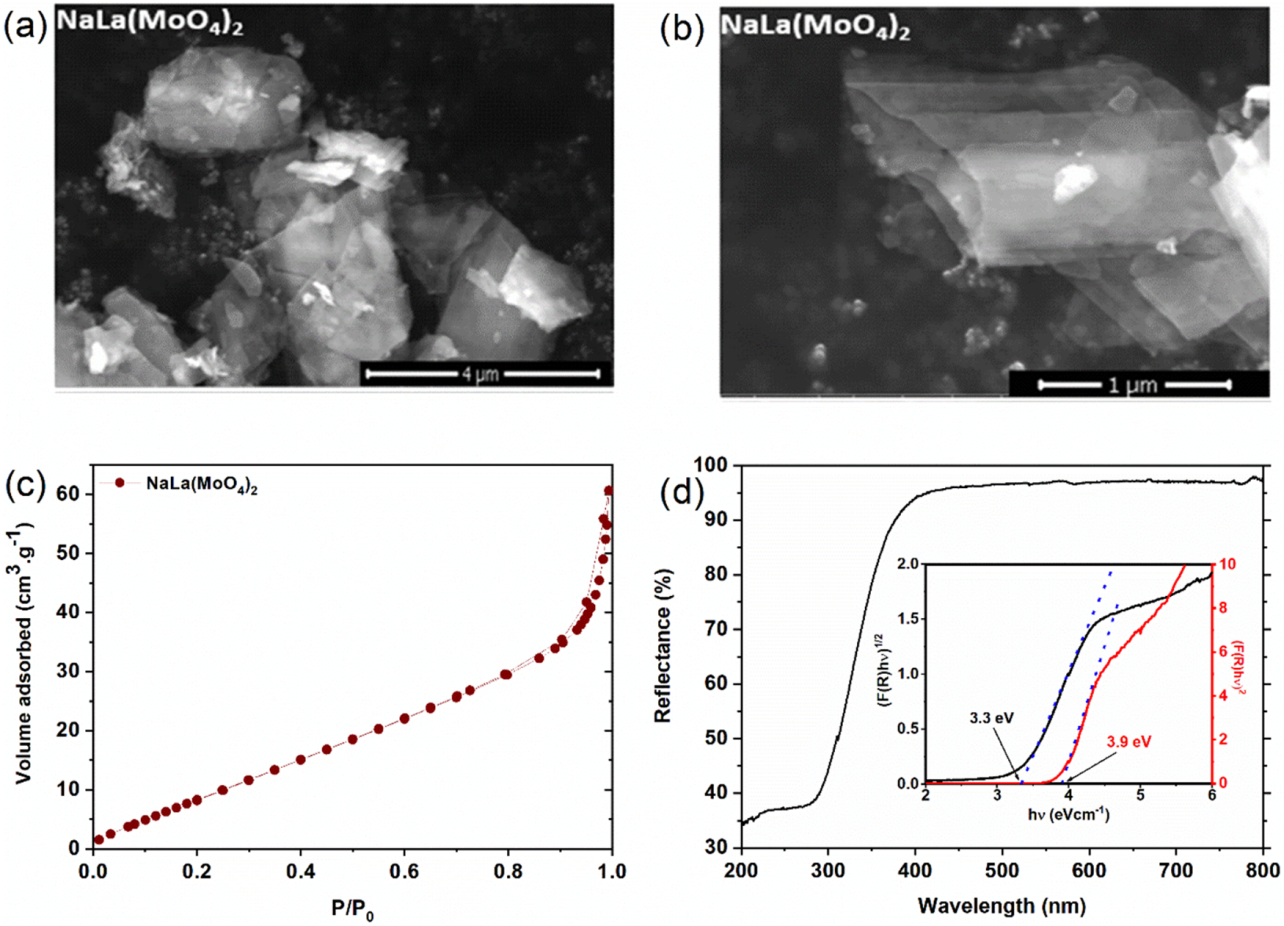
SEM images of NaLa(MoO_4_)_2_ viewed at (a) 10,000× and (b) 30,000× magnification; (c) its nitrogen adsorption/desorption isotherms; and (d) UV–vis transmission spectra and prospection of the *E*_*gap*_ value estimated by the Kubelka–Munk method (inset).

Figure 3a,b illustrates SEM images that elucidate the morphology of NaLa(MoO_4_)_2_. It is observed that the molybdate crystals exhibit an overlapping plate-like morphology, displaying an irregular shape and size. This observation is consistent with the findings of Zhang et al.,^32^ who synthesized nano- and microcrystals of pure and M-doped NaLa(MoO_4_)_2_ (M = Er^3+^, Nd^3+^, and Yb^3+^) by the microwave-assisted hydrothermal method, using different concentrations of La(NO_3_)_3_ and varying the temperature conditions. The use of 0.02 mol L^–1^ La(NO_3_)_3_ between 100 and 120 °C resulted in the observation of materials in the form of bipyramid. At temperatures between 140 and 160 °C, the crystal assumes a dendritic morphology. At 180 °C, molybdates were observed to take the form of rods, dendrites, and ellipsoids when La(NO_3_)_3_ concentrations were 0.01, 0.03, and 0.05 mol L^–1^.

Figure 3c depicts the nitrogen adsorption/desorption isotherms for NaLa(MoO_4_)_2_, which exhibit a curve analogous to that of the Type II isotherm, as defined by the International Union of Pure and Applied Chemistry (IUPAC). Type II isotherms are typically observed in materials comprising plate-shaped particles, exhibiting the presence of micropores (≤2 nm), mesopores (>2 and ≤ 50 nm), and macropores (≥50 nm).^33^ These findings are consistent with those observed in the SEM images, which revealed that NaLa(MoO_4_)_2_ exhibited a plate-to-plate overlap structure. The BET and BJH algorithm methods were employed to determine the surface area and pore diameter of NaLa(MoO_4_)_2_, the resulting values were 45.71 m^2^ g^–1^ and 6.54 nm, respectively. Thus, the synthesized material can be classified as mesoporous, with a diameter ranging from 2 to 50 nm. These findings may positively influence the photocatalytic reactivity of the material, as a greater number of organic pollutant molecules may be adsorbed on the porous surface, thereby enhancing its efficiency in dye degradation.

Figure 3d depicts the UV–vis transmission spectra, which exhibit a transmittance of 96% at wavelengths exceeding 500 nm. Furthermore, the inset illustrates the bandgap, which was calculated using the Tauc plot relation
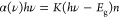
2In this equation, α(*ν*) represents the absorption coefficient, *K* is a constant, *E*_*g*_ denotes the bandgap energy, *hν* is the incoming photon energy, and *n* is 1/2 for direct and 2 for indirect electronic transitions. The direct and indirect bandgap were found to be 3.3 and 3.9 eV, respectively, as illustrated in the inset of Figure 3d.

### 3.3 Photocatalytic Performance

**Figure 4:**
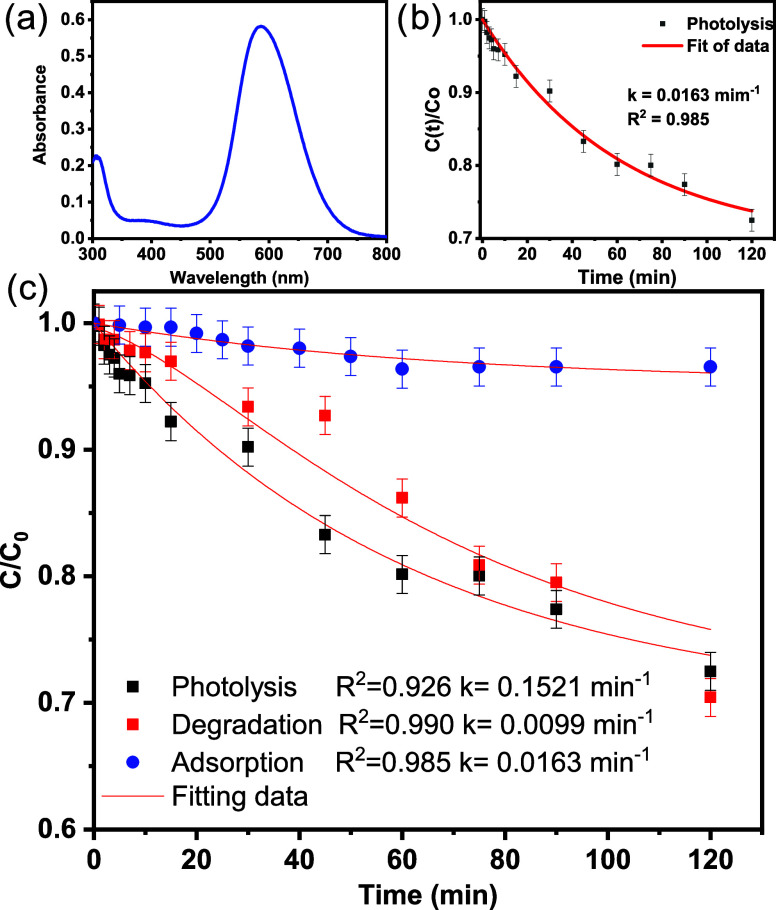
(a) UV–visible spectrum of CBB-250 dye, along with the (b) variation of its concentration along the time under isolated conditions of photolysis (b, c), adsorption (c), and direct photodegradation (c). The following experimental conditions were used: 0.2 g L^–1^ photocatalyst (precursor Na_2_MoO_4_.2H_2_O); 3.5 × 10^–5^ mol L^–1^ starting dye concentration; 303 K temperature; and 270 nm ultraviolet radiation.

The photochemical reactivity of NaLa(MoO_4_)_2_ was evaluated in the context of the CBB-250 dye degradation process. The visible spectrum of CBB-250 (Figure 4a) revealed the presence of absorption bands at 307 and 585 nm, which are associated with electronic transitions occurring in the aromatic ring and chromophore group, respectively. The same spectral profile was employed to assess alterations in dye concentration via photolysis (degradation initiated by light), adsorption (chemical interaction with the surface), and photodegradation using the precursor Na_2_MoO_4_·2H_2_O (mineralization by direct chemical oxidation), as illustrated in Figure 4b, c. The adsorption of the dye on the ceramic material was insignificant, whereas photolysis and degradation demonstrated pronounced and equivalent reactivity. This indicates that both effects are effective in oxidizing the compound in question.

**Figure 5:**
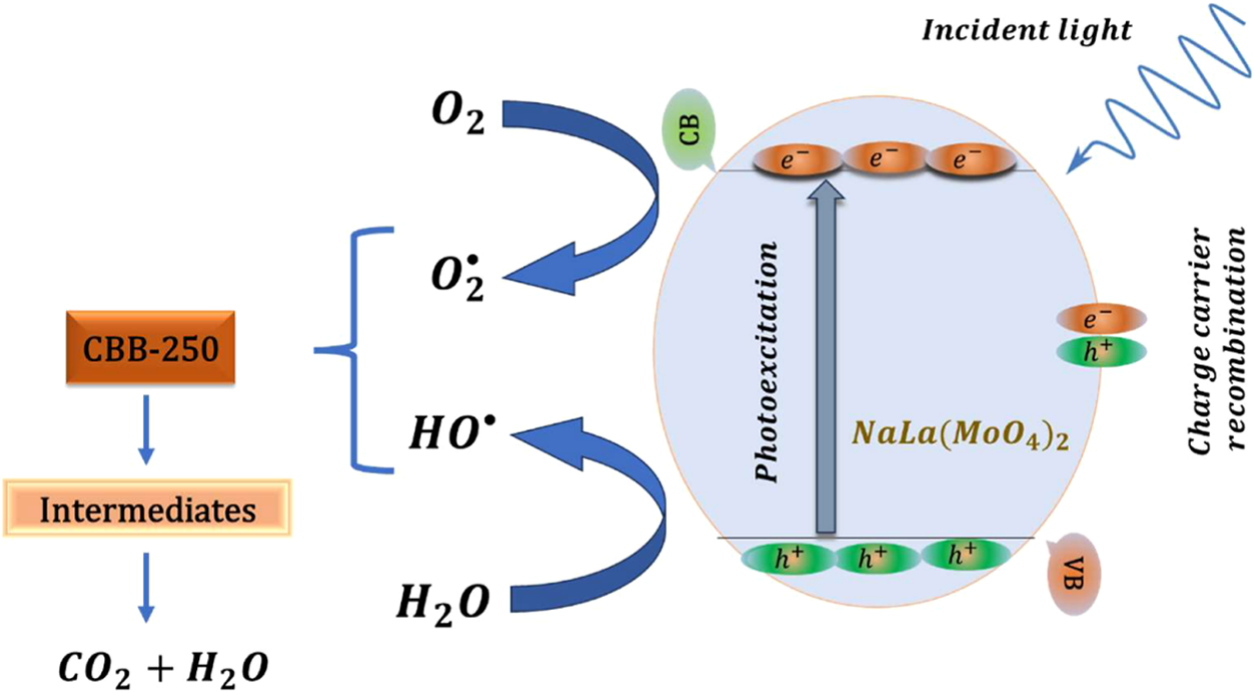
Proposed mechanism for the photodegradation of CBB-250 mediated by NaLa(MoO_4_)_2_.

CBB-250 dye is characterized by its substantial molecular size and functional groups, which predominantly interact with the photocatalyst through physical adsorption mechanisms, such as van der Waals forces or hydrogen bonding, as opposed to the formation of robust chemical bonds.^34^ These weaker interactions serve to minimize the potential for significant perturbations in the semiconductor’s energy levels. Furthermore, the dye functions as a substrate for oxidation by reactive species, such as hydroxyl radicals or superoxide, which are generated by the semiconductor under irradiation.^35^ This process leads to the complete mineralization of organic matter into carbon dioxide (CO_2_) and water (H_2_O) over time. A schematic representation of this mechanism is provided in Figure 5. The predominantly external interaction ensures that the energy levels of the semiconductor remain largely unaffected, which also accounts for the consistency observed across various photodegradation cycles.

The observed pH variations in the solution are consistent with the findings reported by Chial et al.^36^ Their study demonstrated that the formation of a complex with CBB-250 stabilizes the negatively charged anionic form of the dye, resulting in a blue color even under acidic conditions, where most molecules in the solution are in the cationic form. Suppression of the dye’s absorption band under acidic conditions was similarly observed (Figures S1a and S1b).

Figure S1c presents the pH variations during the adsorption and degradation processes of the dye over time. During the adsorption process, a slight decrease in pH from 6 to 5 was observed, while during the photocatalytic degradation, a pronounced pH decline from 6 to 3 was evident, followed by a recovery to pH 6 after a 20 min reaction period. This behavior underscores the photocatalytic activity of NaLa(MoO_4_)_2_ under irradiation, highlighting the reaction time required for dye removal or degradation.

Figure S1d provides a visual representation of the absorption and emission spectra of the solution during photocatalysis. Initially, the emission band shifted to longer wavelengths but later returned to its original position. This initial shift led to an overlap between the emission band and the dye’s absorption spectrum, which also underwent suppression during the early stages of the reaction. The intersection of these bands facilitated charge transfer, which was activated by the excitation of the material upon exposure to radiation. The analysis of the emission and absorption spectra (Figure S1d) corroborates the occurrence of the aforementioned overlap, thereby indicating the occurrence of energy transfer between the nanoparticle and the dye.

At this juncture, two potential energy transfer mechanisms can be considered: dipole–dipole interactions or charge transfer processes (Förster or Dexter mechanisms). The adsorption process depicted in Figure S1d supports the formation of a nanoparticle-dye complex, which suggests a predominance of Dexter-type processes in this system.^37^

**Figure 6:**
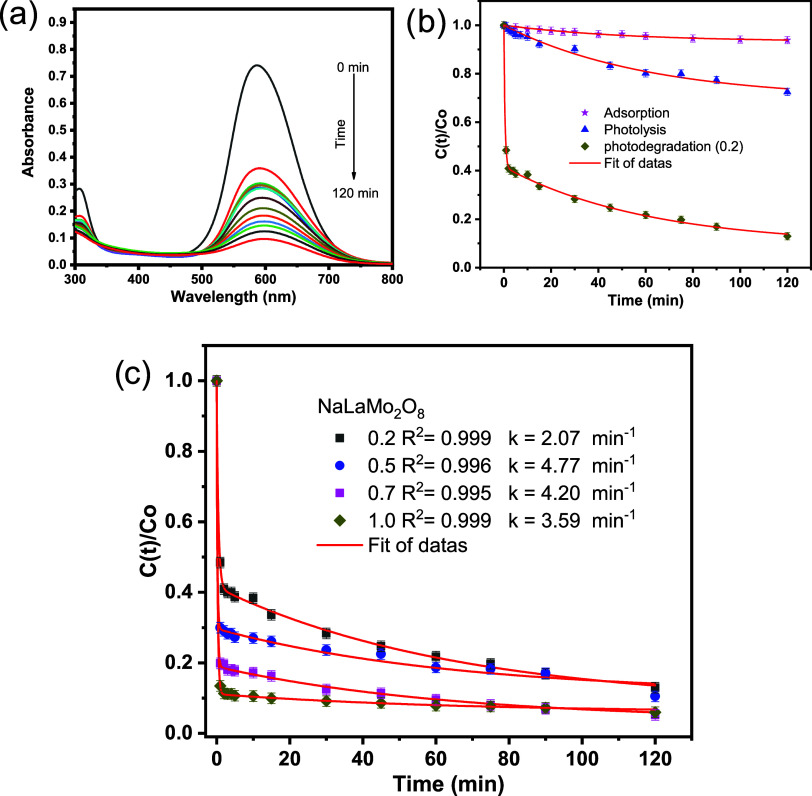
(a) UV–visible spectrum of CBB-250 photodegradation as a function of photodegradation time using NaLa(MoO_4_)_2_ under irradiation. The following experimental conditions were used: 0.2 g L^–1^ photocatalyst; 3.5 × 10^–5^ mol L^–1^ starting dye concentration; 303 K temperature; and 270 nm ultraviolet radiation. (b) Variation of CBB-250 concentration with time, monitored under optimized conditions of photolysis, degradation, and photodegradation. (c) Effect of changing photocatalyst concentration on reaction kinetics.

The kinetic constant in each process can be estimated by solving the following differential equations
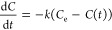
3
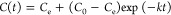
4In this context, *C*_0_ represents the initial concentration of the compound, *C*_e_ is the equilibrium concentration, *C*(*t*) represents the concentration of the pollutant at the conclusion of the process, and *k* is the rate constant associated with the pseudo-first-order reaction. Upon substituting the experimental data, the value of *k* was observed to vary from 0.0099 to 0.0163 min^–1^ (*R*^2^ = 0.985). The experiments were conducted at a pH of 7.0 to maintain the structural and functional integrity of the photocatalyst. Exploration of typical pH variations was avoided, as acidic or basic conditions have been demonstrated to induce dissolution, leaching, and precipitation reactions that compromise the photocatalyst’s stability, thereby diminishing its activity and long-term usability. The following section delineates the potential reactions that could contribute to such degradation
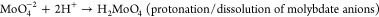
5
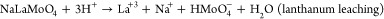
6
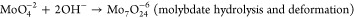
7
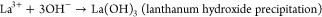
8With regard to the combined impact of photolysis and oxidative degradation (i.e., photodegradation), experiments were conducted with CBB-250 in the presence of molybdate dispersed in solution and under irradiation. The results of the spectral variations over time are presented in Figure 6a. The absence of band displacement indicates that the dye underwent gradual mineralization over time during the course of the treatment. Furthermore, it was observed that the rate of decay of the molecule’s absorption bands is significantly faster under photodegradation conditions (>95% oxidation) compared to those obtained when the degradation and photolysis (<30% oxidation) phenomena were used separately (Figure 6b). This indicates a positive synergistic effect. It is well established that the photodegradation process of many ceramic materials is due to the generation of ^•^OH radicals, which are formed by the interaction of these materials with incident radiation. This makes them more reactive to the oxidation of water molecules. NaLa(MoO_4_)_2_ functions as a light-harvesting agent, accelerating the formation of electron–hole pairs that are responsible for its high reactivity in generating oxidants. These radicals are distributed both in the reaction medium and on the surface of the material.

As the concentration of the photocatalyst was increased from 0.2 to 1.0 g L^–1^, the process kinetics became more critical. The values of k oscillated between 2.07 and 4.77 min^–1^, as illustrated in Figure 6c. Table 1 provides a summary of the photocatalytic reactivity of NaLa(MoO4)_2_. The results demonstrate that the material exhibits significant adsorption, achieving a dye removal rate of 30%. The efficiency is excellent, with *Q*_eff_ exceeding 85%. However, there are *K* values (∼4.7 min^–1^) for the 0.5 and 0.7 g L^–1^ catalyst concentrations. Furthermore, as previously stated, NaLa(MoO4)_2_ exhibits a high surface area, which enables an enhanced interaction with light and reduces the recombination of charge carriers. The investigated molybdate demonstrated effective photocatalytic capabilities for the degradation of the CBB-250 dye, indicating its potential as an alternative for the treatment of organic pollutants.

**Table 1:** Summary of the Photocatalytic Reactivity of NaLa(MoO_4_)_2_ in Degrading the CBB-250 Dye

system		K (min^–1^)	Ce(±0.01)	*Q*_eff_ (%)	adsorption (% ± 0.01)
photolysis		0.0163 ± 0.002	0.69	30.60	---
Na_2_MoO_4_·2H_2_O (g/L)	0.2	0.0099 ± 0.001	0.70	29.80	4.70
NaLa(MoO_4_)_2_ (g/L)	0.2	2.07 ± 0.1	0.12	87.67	6.78
	0.5	4.77 ± 0.1	0.13	86.28	16.96
	0.7	4.20 ± 0.1	0.041	95.89	23.75
	1.0	3.59 ± 0.1	0.062	93.75	33.93

Titanium dioxide (TiO_2_), a material of significant interest in the field of photocatalysis, has been widely recognized as a primary agent in the degradation of organic compounds in textile effluents. Its photocatalytic properties have been particularly effective in the degradation of dyes, such as CBB-250. Bukallah et al.^34^ reported the photocatalytic discoloration of CBB-250 using anatase-phase TiO_2_, achieving 51% dye removal within 30 min. In comparison, the results presented in this study demonstrate that NaLa(MoO_4_)_2_ exhibits superior catalytic performance compared to that of TiO_2_.

## 4 Conclusions

The coprecipitation method is a strategic approach for the production of NaLa(MoO_4_)_2_ by microcrystals with a tetragonal arrangement. These microcrystals exhibit plate-to-plate overlap, which attenuates the surface energy of the structures. Furthermore, the physicochemical characterizations indicate that the material produced exhibits a mesoporous nature, with a high surface area, and is sensitive to UV light. This makes it an intriguing candidate for facilitating the reaction kinetics in a heterogeneous photocatalysis process. In this study, the photocatalytic efficiency of the produced molybdate was demonstrated through degradation tests of CBB-250 with an oxidation rate of 95.9% achieved within a 2 h treatment period. In light of the pressing need for alternative materials to treat emerging pollutants in potentially compromised matrices, NaLa(MoO_4_)_2_ presents a compelling option for incorporation into assistive technologies.

## Supporting Information Available

UV–visible spectrum of CBB-250 and degradation efficiency at different pH values; pH variation as a function of adsorption and photodegradation processes; and absorption and emission spectra recorded during the dye photodegradation process (PDF)
