# Supplementary material for: Preparation and Application of Sodium–Lanthanum Molybdate for the Photocatalytic Degradation of Coomassie Brilliant Blue G-250 Dye
Source: ACS Omega. 2025 Apr 17;10(16):16006–14. doi: 10.1021/acsomega.4c08777 (PMC12044509; doi:10.1021/acsomega.4c08777)
Supplement: Supplementary file 1 — ao4c08777_si_001.pdf [file ao4c08777_si_001.pdf]

Supporting Information

Preparation and application of sodium-lanthanum molybdate for photocatalytic degradation of Coomassie Brilliant Blue G-250 dye

Caique D. A. Lima [1,2], Joyce A. Borges [3,4], Italo A. L. Santos [1], Angel A. Hidalgo [1], Josy A. Osajima [3], Adriel da Silva Almeida [5], Thiago M. B. F. Oliveira [5], Jefferson F. D. F. Araujo [2], Suellen D. T. de Barros [2], Marcelo E. H. Maia da Costa [2], Diego A. B. Barbosa [6], João V. B. Moura [6], Gardênia S. Pinheiro [1], and Cleânio L. Lima [1,*]

[1]*Department of Physics, Campus Universitário Ministro Petrônio Portella, Universidade Federal do Piauí, Bairro Ininga, Teresina, PI CEP: 64.049- 550, Brazil.*

[2]*Department of Physics, Pontifical Catholic University of Rio de Janeiro, Rua Marques de São Vicente, 22451-900, Rio de Janeiro, Brazil.*

[3]*Laboratório de Materiais Avançados – Limav, Campus Universitário Ministro Petrônio Portella, Universidade Federal do Piauí, Bairro Ininga, Teresina, PI CEP: 64.049- 550, Brazil.*

[4]*Department of* Chemistry*, Campus Universitário Ministro Petrônio Portella, Universidade Federal do Piauí, Bairro Ininga, Teresina, PI CEP: 64.049- 550, Brazil.*

[5]*Science and Technology Center, Federal University of Cariri, Av. Tenente Raimundo Rocha, 1639, Cidade Universitária, Juazeiro do Norte 63048-080, CE, Brazil.*

[6]*Department of Physics, Federal University of Maranhão, 65080-805, São Luís, MA, Brazil.*

*Corresponding author: (Cleânio L. Lima, PhD)
E-mail address: cleanio@ufpi.edu.br

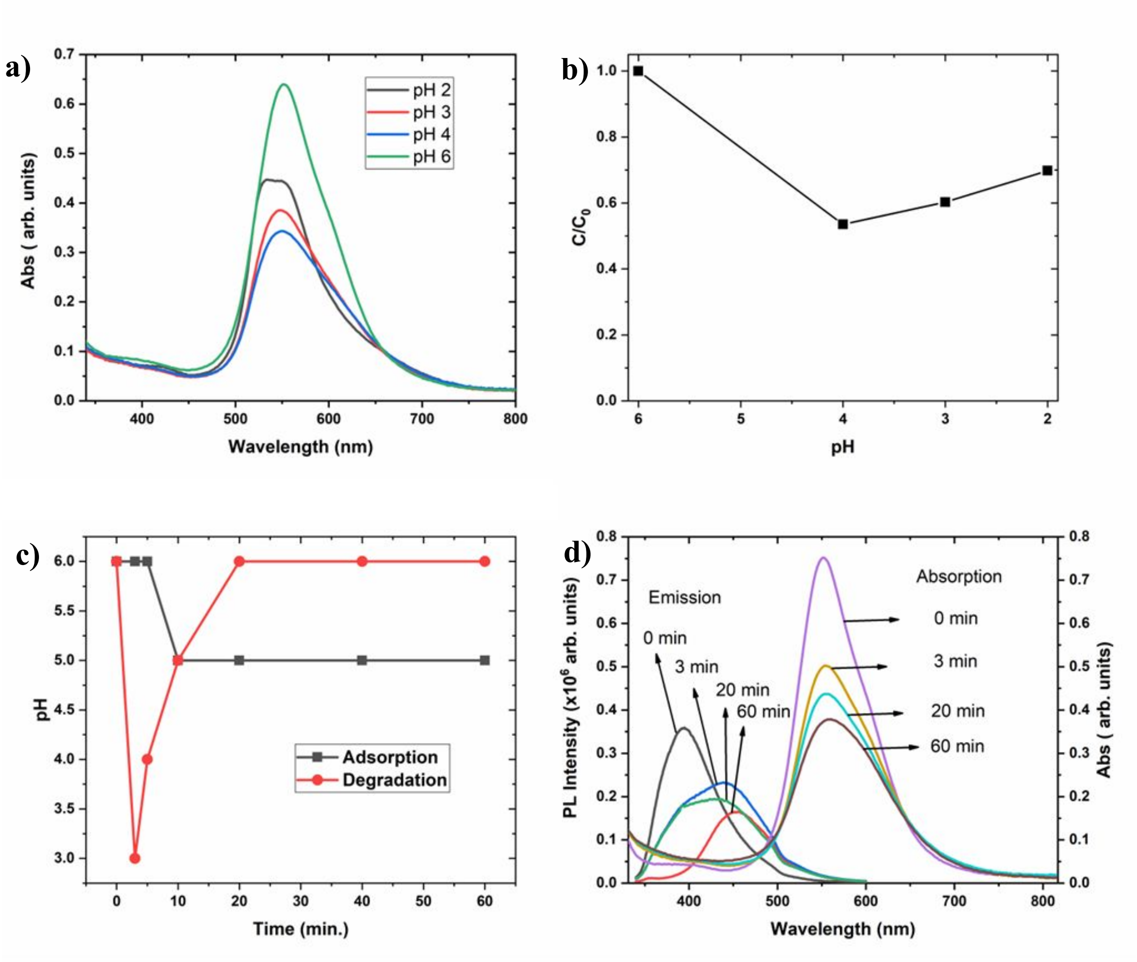

Figure S1: a) UV-Visible spectrum and b) degradation efficiency of CBB-250 at different pH values, c) pH variation as a function of adsorption and photodegradation processes, and d) emission and absorption spectra during the photodegradation process.
